# Exploiting Plant–Phytonematode Interactions to Upgrade Safe and Effective Nematode Control

**DOI:** 10.3390/life12111916

**Published:** 2022-11-17

**Authors:** Mahfouz M. M. Abd-Elgawad

**Affiliations:** Plant Pathology Department, Agricultural and Biological Research Institute, National Research Centre, El-Behooth St., Dokki, Giza 12622, Egypt; mahfouzian2000@yahoo.com

**Keywords:** nematode management, biocontrol, mechanisms, plant–nematode interactions, optimizing strategies

## Abstract

Plant-parasitic nematodes (PPNs) bring about substantial losses of economic crops globally. With the environmental and health issues facing the use of chemical nematicides, research efforts should focus on providing economically effective and safe control methods. The sound exploitation of plant-PPN interactions is fundamental to such efforts. Initially, proper sampling and extraction techniques should be followed to avoid misleading nematode data. Recent evolutions in plant-PPN interactions can make use of diverse non-molecular and molecular approaches to boost plant defenses. Therefore, PPN control and increasing crop yields through single, sequential, dual-purpose, and simultaneous applications of agricultural inputs, including biocontrol agents, should be seriously attempted, especially within IPM schemes. The use of biologicals would ideally be facilitated by production practices to solve related issues. The full investment of such interactions should employ new views of interdisciplinary specialties in the relevant modern disciplines to optimize the PPN management. Having an accurate grasp of the related molecular events will help in developing tools for PPN control. Nonetheless, the currently investigated molecular plant-PPN interactions favoring plant responses, e.g., resistance genes, RNA interference, marker-assisted selection, proteinase inhibitors, chemo-disruptive peptides, and plant-incorporated protectants, are key factors to expanding reliable management. They may be applied on broader scales for a substantial improvement in crop yields.

## 1. Introduction

Optimizing the approaches and compounds utilized to manage plant pests/pathogens via more reliable and safer techniques represents a current pressing challenge for sustainable agricultural systems. The dissatisfaction with synthesized pesticides, such as chemical nematicides, has been increasing. These trends concerning a lack of content for numerous pesticides are emanating from their unfavorable impacts, e.g., their unacceptable influences on ecological contamination and human health, non-target beneficial organisms, and the development of resistance-breaking pathotypes [1,2,3]. Thus, the successes of the alternative techniques are mainly anchored in the available chances to phase out such unsafe chemicals via introducing environmentally benign products.

Challenges to conventional plant-parasitic nematode (PPN) control procedures are more evident than before, given the current huge assembly of knowledge on plant-PPN interactions to be properly exploited. Furthermore, such a case has obviously disclosed the limitations of conventional studies on plant–nematode interactions and showed an urgent need to search for new interdisciplinary specialty views for optimal integrated pest management (IPM) to reduce the worldwide food deficit gap [4,5]. Interestingly, various approaches and techniques are continuously evolving to address and obtain the use of plant–nematode interactions, but it is still generally difficult to find a promising PPN control tactic that is economical, safe, and effective. Therefore, this review sheds light on the yield losses caused by PPNs and their common but imperfect management methods as a background to demonstrate the importance of continuing to improve PPN control measures. It aims to focusing on the main progress in the current studies of plant–nematode interactions from the standpoint of optimizing PPN management. This review also discusses emerging molecular techniques in nematology to harness the study of mechanisms underlying the positive aspects of plant–nematode interactions to boost PPN management. To avoid confusion, the term plant-parasitic nematode may differ somewhat from the term plant nematode (or phytonematode), but what is meant by both is the former term (PPN).

## 2. Nematodes, Crop Losses, and Their Control Methods

The role of PPNs in causing economically significant yield losses is steadily increasing as we become aware of their wide spread, complex diseases, invasive and quarantine species, and the emergence of resistance-breaking races/strains. Being obligate parasites, PPNs must feed on plant parts, mostly roots, but some species parasitize aerial plant parts to grow and reproduce sexually or asexually (without fertilization). The nematodes parasitizing aerial parts include the foliar nematodes, seed gall nematodes, and stem and bulb nematodes. Second-stage nematode juveniles (J2s) hatch from eggs, mostly in the infective stage. While J2s typically parasitize the plant roots of their hosts (they are occasionally attracted to the plant via root exudates), immune/non-host plants may repulse the nematodes by repellent compounds in their roots. Thus, plant–nematode interactions display a variety of characteristics and may actually start before the PPN touches the plant roots [6]. Based on the nematodes’ feeding habits, PPNs are separated into the following major groups: (1) Ectoparasites: They are primitive parasitic forms. Species of the genera *Helicotylenchus*, *Tylenchorhynchus*, *Xiphinema*, and *Longidorus* are examples of these forms, as they have the ability to penetrate the plant root tissues with the help of their stylets [7]. They remain outside the roots but feed on their epidermal or subepidermal tissues. All PPNs have the basic attributes of inserting their stylets into the cells of their host tissues, injecting secretions, and taking the cell contents out. However, each species has its own feeding behavior, and consequently each causes a specific mode of tissue damage [6]. (2) Migratory endoparasites: They usually penetrate and migrate within the roots for feeding, e.g., *Ditylenchus dipsaci* and *Pratylenchus* spp. They can even leave a rootlet to penetrate another one. Both migratory endoparasites and ectoparasites grow and molt to J3, J4, and lastly mature with no sexual dimorphism. (3) Semi-endoparasites: They utilize only their head to enter the root, but the posterior part remains in the soil, e.g., *Rotylenchulus reniformis* and *Tylenchulus semipenetrans* [8]. Therefore, they must settle at specific sites on the roots. They have sexual dimorphism since the female body swells outside the roots. (4) Sedentary endoparasites, e.g., cyst nematode (CN) species (*Globodera* and *Heterodera* spp.) and root-knot nematode (RKN) species (*Meloidogyne* spp.). Their J2s move into and set themselves within the roots, where the developed females form swollen sedentary parasites and exhibit sexual dimorphism. They sometimes protrude on the root surface. Conceivably, sedentary and migratory endoparasites can bring about more damage in the root tissues than ectoparasites as they invade, migrate, and feed within their plant hosts. Investigations concerning plant–nematode interactions have mostly focused on both cyst and root-knot nematodes due to the widespread and substantial yield losses that these sedentary parasites inflict on global agricultural production. The initiation and development of sedentary-nematode feeding sites for RKNs are distinct from those of CNs. The J2s of RKNs organize their feeding sites at the root-differentiating vascular tissue in the form of distinguished nurse/giant cells. The J2s of CNs form their feeding sites by establishing syncytia (a few cells are combined as their walls are resolved) near the vascular bundle. Having established these sites to enable nematode feeding on the cell contents, the nematodes (CN or RKN) develop until maturing into females. These nematodes lay eggs that hatch new J2s. Ultimately, PPNs can variably interact with their susceptible plants, ranging from the primitive form of the ectoparasites to the close association of sedentary endoparasites within the roots of their hosts [9].

Global losses in crop production due to phytonematodes for the 20 life-sustaining crops was valued at 12.6% of the total crop yield. This percentage reflected USD 215.77 billion in annual worldwide crop losses. Moreover, a second group of 20 crops with considerable values for human food and commerce have a 14.45% annual global loss in crop production, i.e., USD 142.47 billion. Together, these 40 important crops have about 13.5% losses worth USD 358.24 billion each year [10]. Obviously, these estimates will probably be raised via additional PPN-infected plant species in the long list of inflicted crops. Both RKNs and CNs contribute the lion’s share to bringing about these losses.

Presently, various tactics and strategies use a variety of materials to manage nematodes. These comprise chemicals, biocontrol agents, resistant plant genotypes, soil solarization, the use of certified plant materials, and cultural practices such as crop sequencing, deep summer ploughing, flooding, fallowing, and soil amendments [11]. As most PPNs live in the rhizosphere soil or within plant roots, the nematicidal contact of the related chemicals to these surroundings is generally problematic [12]. This difficulty does not negate the effective suppression of PPN populations via chemical nematicides, e.g., [13]. Conversely, there is a growing dissatisfaction with these chemicals due to their potential threat to human health, environmental pollution, and the development of resistance-breaking PPN races/strains. Such issues have directed many researchers to seek reliable but safe alternatives. Most bionematicides are safe, but they are sometimes slower-acting and more inconsistent than the toxic chemicals [14]. Frequently, the less effective performance of biological control agents (BCAs) and/or their bioactive materials relative to chemical nematicides in numerous situations and their inconsistent control results, ascribed to many biotic and abiotic factors as well as product quality control, are the major impediments to their use in PPN management programs [14,15,16,17]. Crop sequence strategies to manage PPNs would be reliable and safe approaches, were it not for the insufficiency of the PPN immune/resistant plant genotypes/varieties required in the crop rotation. Various soil amendments for arable areas can boost plant growth, although with the possible development of PPN population levels and/or the existence of harmful nematodes and other pathogens in the added materials, exceptions should be anticipated [15]. On the contrary, such additions containing plant extracts/botanical matrices and purified secondary metabolites are continuously progressing [16], but costs, registration procedures, and time-consuming problems are slowing their adoption [18]. Dutta et al. [2] recommended economic benefits, reliability, and safety as prerequisites to utilize these BCAs and their compounds. Fallowing and flooding are sometimes utilized for nematode control but are not usually economical; fallowing leads to periods of unexploited arable soil, and flooding consumes a lot of water. Tillage is generally useful against numerous pests/pathogens, including subterranean nematodes, but can also harm many PPN-antagonistic organisms. Thus, tillage may facilitate increased plant damage by nematodes [15]. Although PPN-free transplants via certified seedlings are excellent strategies, these plants should not develop into PPN-infested soils, which is generally an inevitable action. Ultimately, the aforementioned PPN chemical, biological, and cultural control tactics are not perfectly successful or fully accepted. They are in dire need for deep revisions for reliability and/or safety [19]. Moreover, their disadvantages are sometimes discouraging with reference to the generally low accuracy and precision in sampling the nematodes’ life stages in soil and within plants as well as the aggregated distribution patterns and broad host ranges of most PPN species [20].

Therefore, some authors [21] have recently confirmed that difficulties are apparent in recommending an advantageous PPN management method that is economical, reliable, benign to non-targets, and generally safe. Admittedly, growers/stakeholders can turn to resistant genotypes/cultivars and agricultural practices to manage nematodes. However, for many plant species, these resources and techniques are frequently unavailable or inappropriate. On the contrary, a greater understanding of plant–nematode interactions may provide new and sound tactics/strategies and resources to optimize nematode control. The current progress in investigating such interactions can make use of diverse non-molecular and molecular methods to enhance plant defenses against nematodes and consequently increase crop yields. Therefore, the full spectrum of these interactions should be addressed to alleviate the plant damage caused by PPNs.

## 3. Role of Sampling and Extraction Methods in Grasping Plant–Nematode Interactions

**Figure 1:**
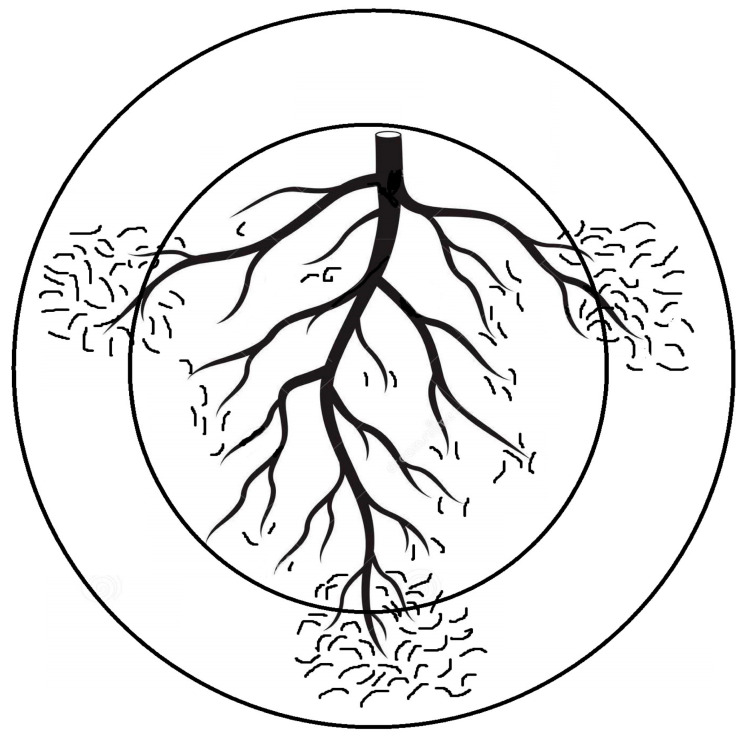
Two quadrat sizes of augers are illustrated by concentric circles. The inner circle displays random nematode distribution around the main plant roots, but the outer circle shows aggregated nematode distribution around lateral fibrous roots [20].

Optimizing sampling and extraction methods for PPNs is fundamental for obtaining a sound understanding of these interactions and exploiting them in order to upgrade benign and reliable nematode control. Because sampling is closely associated with the study of every plant–nematode interaction and consequent PPN management, its possible flaws will negatively affect all relevant scopes. Basically, PPN sampling aims to detect, identify, and assess their population levels in soil/plant tissues. The timing, tool, intensity, pattern, and the related material that is sampled all rely on the intended objective and well-conceived scenario to preclude issues of inaccurate/imprecise sampling and the limitation of available funds [20]. Interestingly, heavily PPN-infected plants may lead to an insufficient root system that cannon support numerous PPNs, but samples taken from nearby less infected roots may host more PPNs due to their comparatively large root system. This situation may disrupt the negative correlation usually assumed between crop yield and PPN population levels. Such a disruption might occur when soil samples obtained from the plant rhizosphere are utilized to assess the PPN number per unit (weight in g or volume in cm^3^). In order to avoid this issue or misleading results, it is advised to count the PPN number per g of fibrous roots in the same soil volume. Otherwise, the discrepancy in the PPN population levels correlated to plant damage may account for a false correlation between the PPN population levels and the growth parameters/productions of infected plants when either weight unit or volume, but not both together, is used. Likewise, using variable samplers for similar integrated pest management (IPM) plans may result in erratic data. Abd-Elgawad [20] elaborated that the area/volume of the sampling units can impact the resulting distribution patterns of the encountered nematodes. Though commonly acceptable, sampling for similar goals is carried out with cores or augers that vary from one trial/location to another. This may also lead to the misinterpretation of data and erratic results. Sampling the same spot with two concentric circles (representing two core diameters) might unintentionally display two different patterns of spatial distribution for the same nematode population (Figure 1). Consequently, counts of the targeted nematode species would require square root (for random distribution) or log (for aggregated distribution) data transformations to stabilize the treatment variances of experimental variables, which is a prerequisite to fulfill the assumptions of parametric statistical techniques, e.g., correlation, regression, and analysis of variance [22]. Moreover, a standardized sampler can provide a reasonable comparison of different experiments and allow the analysis of various individual tests in order to draw substantial and sound conclusions of plant-PPN interactions. To summarize, a 2 cm diameter sampler with adjustable depths was suggested unless the experiment calls for a specific experimental objective [20]. Moreover, the PPN vertical distribution is shaped differently based on the root systems of the host plants. Deep-rooted plants need deeper sampling. For instance, PPNs of grape roots (60 cm root depth) have vertical distribution patterns different from shallow-rooted ones such as squash (20 cm root depth). However, a 30 cm root sampler can generally target the PPNs at their highest and effective densities [20]. Such suggestions avoid other flaws, as the attributes of a distribution pattern mostly rely on the “standard” scale over which it is set. Therefore, relevant suppliers/manufacturers would preferably cooperate with pest control specialists to form/standardize their tools to better configure plant-PPN interactions and IPM schemes. Meanwhile, researchers should use multiple indices of dispersion to supplement each other in order to clarify such interactions and show more aspects of the distribution patterns. Using two indices together, Gorny et al. [23] could set sound sampling schemes and define certain sites to reliably and inexpensively apply nematicide.

The pros and cons of each PPN extraction method should be considered. Sieving and centrifugation utilizing a sucrose gradient may extract and quantify both dead and live PPNs found in soils. On the contrary, the Baermann funnel technique and its modifications can only separate and extract live nematodes. Clearly, additional tests/bioassays may be needed to find the most reliable extraction technique that is closely related to the examined fauna and flora to study certain plant–nematode interactions [24]. As classical extraction methods inherit imperfect extraction efficiency, a series of extractions via the aforementioned techniques to significantly raise the PPN separation efficiency could reasonably alleviate this issue [20,25].

The sampling and extraction of molecular materials and biochemicals related to PPNs are relatively novel approaches. Relevant assays [25] may extract isozymes or proteins from the PPNs, e.g., for identification, or from their plant hosts, e.g., for determining the enzyme activity of a host genotype/cultivar to explore its incompatible or compatible reactions to PPN infection. These procedures to study plant–nematode interactions may designate susceptible/resistant plant genotypes and detect the role of BCAs in priming the colonized roots against PPNs [26]. Furthermore, sampling techniques to define and assess volatiles in situ in the soil atmosphere can facilitate the exploration of the chemical cues responsible for communication among the different trophic levels of the present organisms. Therefore, they can contribute to not only characterizing plant–nematode interactions but also to conceiving the best IPM program [27]. However, favorable outputs can often be attained with PPNs at a particular developmental stage [20].

## 4. Exploiting Various Aspects of Plant–Nematode Interactions for PPN Control

### 4.1. General Aspects of Plant–Nematode Interactions

As the nematodes and their host plants co-evolve in nature, their related genes have presumably balanced co-existence for functioning via the offensive and defensive interactions exerted by the PPNs and the attacked plants, respectively. Although PPN reproduction is possible only if metabolism is shifted to enable nutrient uptake from plant cells, the processes of nematode feeding comprise various morpho-histological, physiological, and biochemical aspects [6]. These processes, as well as structural and molecular depictions of the key events that are involved in the plant-PPN interactions, should be accurately explored for better nematode management. Histopathological, physiological, and biochemical changes have a wide range since the mode of PPN parasitism has presumably evolved from ectoparasite to endoparasite. Thus, the basic disease triangle—that plant harm happens only when a susceptible genotype and its PPNs co-exist in a convenient environment—displays aspects that are focal to numerous techniques to expand the utilization of these different interactions to the favor of the host. Within this broad concept of the interactions, all the aforementioned aspects should be exploited for nematode control as best we can. Clearly, the lesser damage generally caused by ectoparasites compared to endoparasites should not be neglected. Current and emerging approaches to control these ectoparasites should be earnestly tested. Thus, fundamental to the above-mentioned controlling methods, they should be quarantined to block their spread to other areas. Other prophylactic and curing measures to manage these nematodes, in addition to the endoparasitic nematodes, have been generally reviewed [28]. Moreover, specific measures for PPN control have recently been reviewed on important crops such as tomato [29], pepper [30], potato [31], eggplant [32], strawberry [33], and citrus [34].

### 4.2. Exploiting Plant–Nematode Molecular Interactions for Endoparasitic Nematodes

On the other hand, intensified and sophisticated control measures are being developed for the most damaging endoparasitic sedentary nematodes, and to lesser degree migratory nematodes, as the molecular events involved in their interactions with host plants could be soundly explored e.g., [15,16,17,18,19,28]. The interactions of host transcriptomes with infections of many related PPN species are being massively researched [17,33,34,35,36,37,38], mostly focusing on species of RKNs and CNs [39,40,41,42,43]. The bases of such studies are to improve scientific assumptions for better understanding and prediction of these interactions. However, the current review would rather address how to exploit these interactions for nematode control, focusing on the molecular control of nematodes. This approach does not negate the importance of addressing the fundamental investigations and the related techniques that were developed to harness such interactions for further services in nematode management.

#### 4.2.1. Optimizing Specific Molecular Techniques for Better Nematode Control

In this respect, traditional techniques for the transcriptome analysis of sedentary nematodes are sometimes based on extracting the nematodes from the host tissue before RNA sequencing (RNA-seq) [44]. However, a more useful method is to utilize dual RNA-seq where the roots of the host plants and their invading PPNs are simultaneously sequenced. With the latter method, a PPN effector gene could be discovered, and the transcriptomic datasets between pre-parasitic and parasitic juveniles of *Meloidogyne chitwoodi* on potato were compared [45]. Thus, the dual RNA-seq could generate a sophisticated analysis of the *M. chitwoodi* genes displayed during parasitism as well as the encoded foreseen secreted proteins. Additionally, the superior method [45] could considerably decrease the large list of genes in the *M. chitwoodi* secretome researched by the classical technique [44]. That is because extracting the nematodes from the roots resulted in recording genes that were not linked to parasitism. While it was quite difficult to functionally report ≥ 300 genes via the classical technique, dual RNA-seq was able to efficiently characterize the functions of fewer genes, particularly early in the life stages of the parasite *M. chitwoodi* [45]. Therefore, it can facilitate more service in PPN management.

Other techniques should also make proper use of the structure/nature of PPN feeding tubes for optimal PPN control. The ultrastructure of these tubes indicates that *Meloidogyne* spp., but not *Heterodera* spp., can swallow relatively large transgenic proteins generated by *Bacillus thuringiensis* [46,47]. As a result, transgenic Cry5B and Cry6A proteins were swallowed by and consequently suppressed *M. incognita* development and reproduction in fibrous roots of tomato [47,48]. Conversely, such transgenic plant resistance does not operate for *H. schachtii* infecting plant roots that similarly express the Cry5B protein via *B. thuringiensis*. That is because the 54 kDa Cry6A protein is too big to be swallowed by the cyst nematodes; the orifice of the *H. schachtii* feeding tube is narrow: about 23 kDa [49]. Thus, plant pathologists and stakeholders should be aware of such restrictions in using transgenic Cry proteins for managing serious pathogens such as *H. schachtii*.

Likewise, a variety of diagnostic assays are being developed using the quantitative polymerase chain reaction (qPCR) for the internal transcribed spacer (ITS) of rDNA in numerous nematode species, but relevant flaws should be solved/avoided. For example, the apparent inconsistency of the ITS sequences of the lesion nematodes, *Pratylenchus* spp., could raise the inevitable risk of obtaining false-negative reactions as variations are found among individuals from the same *Pratylenchus* species or false-positive reactions are found for fragments from unidentified species [50]. The latter authors emphasized that inaccurate quantification could also happen because sequences of some genes are present in multiple copies of individual nematode cells. Additionally, the copy numbers of particular genes may differ among the developmental stages of the same nematode species [20]. These and other relevant data should be addressed cautiously, as they may participate in obtaining imprecise or misleading molecular PPN–host relationships [39,40,41,42]. Contrariwise, other bioassays and experimental results proved useful but may need further optimization to upgrade safe and effective nematode control. Admittedly, comparative secretome analyses for various PPN species or strains can define molecules that are closely involved in particular aspects of the nematode disease and/or govern PPN virulence in the invaded host. The perfection of these analyses is mandatory to set an optimized molecular control of PPNs [41].

#### 4.2.2. Exploiting RNA Interference for Favorable Plant–Nematode Interactions

Certain genes engaged in the RNA interference (RNAi) pathways of the nematode species could be favorably selected for nematode control [51]. Because RNAi is used to preclude messenger RNA (mRNA) from its role in protein synthesis, it can consequently block the related gene function. Subsequently, silencing several of these functional genes simultaneously in a combinatorial approach could be more impactful in controlling *M. incognita* on tobacco, *Nicotiana tabacum* [21]. Moreover, an RNAi technique could be exploited to determine certain nematode effectors to manipulate them for the reliable control of the pinewood nematode *Bursaphelenchus xylophilus* [52]. The authors used four *B. xylophilus* isolates that varied in their virulence levels to detect virulence determinants. Four determinants were determined and quantitatively evaluated at the transcript level at different stages, pre-inoculation, 3 days after inoculation (dai), and 7 dai into *Pinus thunbergii* seedlings, via a real-time reverse-transcription polymerase chain reaction analysis. They found considerably elevated transcript levels of the determinants Bx-CAT1, Bx-CAT2, and Bx-GH30 in virulent *B. xylophilus* isolates relative to avirulent isolates at two of the three examined stages, that is, at pre-inoculation and 3 dai. Thus, the comparative analyses indicated that Bx-CAT2 and Bx-GH30 share in the *B. xylophilus* virulence of specific isolates on the susceptible trees. They are significantly involved as nematode effectors in pine wilt disease [52]. Hence, RNAi should be exploited to suppress these effectors, which display themselves as genes responsible for nematode virulence.

Obviously, silencing such effector genes in nematode species that bring about infection and damage of susceptible plants via the RNAi strategy could properly avoid the infection and resulting damage. Following this line of thinking, the RNAi could effectively be used for economically important nematode species [19,21,52,53,54,55,56]. It was applied to silence the *Meloidogyne incognita*-related effector genes that interact with transcription factors of host plants to operate key enzymes for cell wall degradation. These related *M. incognita* genes were suppressed, and consequently cell wall degradation was blocked [55]. Clearly, experimentation to exploit plant–nematode interactions via RNAi was proven to upgrade safe and effective nematode control. Therefore, this technique should be wisely manipulated and expanded on a field scale as long as there are no negative consequences on both the environment and the agronomic performance of the transgenic plants. In this respect, Iqbal et al. [51] recommended that the submission of double-stranded RNA (dsRNA) to the intended nematode species via RNA silencing is more workable than any other method (e.g., spraying) for a PPN management strategy. For the perfection of the related processes, they [51] proposed that the original goal, the resistant phenotypes of nematode species/strains, and the current integration advantages of RNAi should further be researched because some of the effector genes may react to RNAi differently. For quite favorable plant–pathogen interactions, it is preferable that an RNAi technique induces broad-spectrum resistance or at least stable resistance to the intended nematode species, i.e., confirmed in the progenies of the subsequent generations of the transgenic plant genotype [56].

#### 4.2.3. Upgrading the Utility of Resistance Genes

The arsenal of PPN resistance (*R*) genes should be fully exploited and boosted. *R* genes that were already successfully cloned and transferred from definite plant cultivars to susceptible ones (e.g., *Hs1^pro−1^* from *Beta procumbens* against *H. schachtii*, *Hero A* from Solanum lycopersicum against *Globodera rostochiensis*, the *Mi-1.2* from S. lycopersicum against *M. incognita*, *Gpa-2* from *Solanum tuberosum* against *G. pallida* and *G. rostochiensis*, and *Cre* loci from *Aegilops* spp. against *Heterodera avenae* infecting *Triticum aestivum* [41,57]) should be further expanded. Other *R* genes such as *Me* in pepper, *Rk* in cowpea, *Rhg1* in soybean, *Ma* in *Prunus* spp., and *Mex1* in coffee have proven benefits in elevating resistance against *Meloidogyne* spp. [41,58]. Plant resistance to nematodes, as an example to be followed, was acquired in transgenic lettuce, *Lactuca sativa*, as the *Mi-1* gene present in tomato was cloned and transferred to this distant plant species [59]. Clearly, challenges should be faced to extend resistance and its stability and durability via wise the incorporation of proper *R* genes into economic crops [41]. Because *Heterodera glycines* is the major soybean pest in intensively and broadly cultivated areas of the world, a supporting action was adopted to compensate for the diminishing plant resistance displayed by a high copy number of the *rhg1-b* allele. This was materialized via a plant-incorporated protectant, that is, *B. thuringiensis* delta-endotoxin (Cry14Ab) [60]. As a result, Cry14Ab-expressing soybean plants exhibited fewer *H. glycines* cyst and egg counts than control plants with only the *rhg1-b* allele. Such techniques for exploiting plant–nematode interactions for a more favorable support of plant defense could demonstrate the potential of Cry14Ab to control PPNs.

Another challenge is linked to the gene construct itself, e.g., dual or single genes. Genetically engineered tomato plants with PjCHI-1 and CeCPI in double-structured genes and synthetic promoters displayed diminished RKN infection and multiplication compared to their corresponding tomato plants with a single gene [61]. Moreover, the maintenance of durable resistance may be obtained by conveying multiple resistance genes to specific cultivars within IPM programs. Moreover, the recent detection of a nematode-associated molecular pattern (NAMP) plant receptor may be exploited as a useful tool to develop durable plant resistance against economically important PPNs [40,62]. For such programs, safe chemical pesticides, crop rotation, and/or BCAs/their bioactive compounds can aid in decreasing the pressure on resistant plant species/varieties to lessen/avoid the development or emergence of virulent PPN populations.

Likewise, the technique of using an expression quantitative trait locus (eQTL) map to characterize interacting sets of host plant and PPN genes may be harnessed to investigate how the nematode species can fix gene expression differently according to the genetic constitution of the attacked host plant [37]. For better processing, putative targets, defined by Perry and Moens [38], in the PPN life cycles for various life strategies of PPN genera/species should be the focal points for nematode management. Moreover, comparative genomics can increase our knowledge regarding their life cycles, parasitic strategies, and vulnerable life stages. Obviously, further investigation of incompatible PPN-plant interactions should focus on plant species/cultivars with limited resources of available genetic and genomic materials such as commercial full genome arrays, completed genome and transcriptome sequences, and near isogenic and mutant lines [39]. Even with the relatively few detected *R* genes, there are factors that can break their resistance processes. Among these factors, high temperature, virulent RKN populations, and high PPN population densities were recently reviewed [41]. Therefore, plant breeders and nematologists would preferably rely on activating numerous *R* genes or QTLs, not a single gene, to circumvent these shortcomings for the economically important plant species [63,64,65].

Eventually, a variety of *R* genes with various pathways and expressions may cooperate for specific plant phenotypes. Therefore, more knowledge is desperately needed regarding the mechanisms engaged in host-specific resistance/susceptibility formed by PPN effectors and resistance genes or quantitative trait loci to upgrade their reliable and durable use in chief crops. Abd-Elgawad [41] proposed utilizing the effects of these approaches on a case-by-case basis. This will enable biologists to track and improve PPN management based on the existing variables. It would grant us a precise fixing of the factors deciding the gene expressions and functions as well as link them to other PPN control strategies into IPM schemes.

#### 4.2.4. Marker-Assisted Selection to Ease and Perfect Nematode Management

Marker-assisted selection (MAS) is a useful method for both setting the introgression of genes linked to desirable plant traits and favoring the characterization of molecular PPN–host interactions. Therefore, MAS may work in the position of chromosome landmarks and should further be utilized for gene incorporation and stacking. A materialized merit of MAS is its ability to characterize tomato cultivars that possess multiple disease resistance traits. In this example, the Mi-1 homologs can bestow resistance on these cultivars to control a relatively wide range of pests/pathogens. The latter comprise not only the most widespread RKNs (*Meloidogyne incognita*, *M. javanica*, and *M. arenaria*) but also important insects, that is, sweet potato whitefly (*Bemisia tabaci*), potato aphids (*Macrosiphum euphorbiae*), and oomycetes (*Phytophthora infestans*) in tomato plants [66,67]. Thus, a variety of methods, depending on molecular markers for phytonematode resistance, should be developed and effectively utilized to select a wide range of major plant species/varieties for resistance against economically important PPN pests [68,69]. These methods usually include restriction amplified length polymorphisms (RALPs), amplified fragment length polymorphisms (AFLPs), cleaved amplified polymorphic sequences (CAPS), random amplified polymorphic DNA (RAPDs), reverse-transcription polymerase chain reaction (RT-PCR), sequenced characterized amplified regions (SCAR), single-nucleotide polymorphisms (SNPs), simple sequence repeats (SSRs), and sequence-tagged sites (STS). Furthermore, MAS seem to be ready to work, even on a broader genetic pool than the currently limited resistance sources via surveying diverse habitats for novel PPN-*R* genes. Examples of recent progress in marker-assisted breeding programs could cover not only the main diploid crop species but also polyploid plant species such as sweet potato, *Ipomoea batatas* (Table 1). For such a complexity of the polyploid genetic constitution and the highly heterogeneous genome of *I. batatas*, the genome-wide association technique, which utilizes multiple dose markers to evaluate autopolyploid species was developed [70].

#### 4.2.5. Other Techniques to Facilitate Molecular PPN Control

Emerging and novel techniques should be utilized and developed in favor of plants while interacting with nematodes. For instance, utilizing enzyme inhibitor coding genes such as proteinase inhibitors (PIs) can preclude the operation of proteinases/proteases freed from the nematodes while attacking the plant. Such PIs could be activated against all proteinases, metalloproteinases, aspartic, cysteine, and serine of these nematodes. Techniques to exploit PIs against the nematodes were recently reviewed [81,82]. Other types of anti-nematode proteins, e.g., Bt Cry proteins, several antibodies, and lectins, can be used with various modes of action against PPNs in plants. Therefore, their action to suppress the nematodes should be well-directed, as aforementioned, to optimize PPN control e.g., [47,48,49]. Most of these compounds are generated by BCAs. On the other hand, a few chemodisruptive peptides can disrupt the PPN usage of their chemoreceptive neurons to contact their host plants or move away from their non-hosts. These neurons recognize specific chemical stimuli for attacking and evading their host and non-host plants, respectively. In this respect, acetylcholinesterase (AChE) and/or nicotinic acetylcholine receptors are typically utilized for the proper function of their nervous systems. However, certain peptides, usually at low concentrations, are able to bind with these receptors and subsequently disrupt the nematode’s chemoreception ability via blocking their response to chemical signals [82]. A peptide generated by genetically engineered potato plants could suppress *G. pallida*-AchE, leading to the disorientation and misguidance of the nematodes when attacking the host plant. The technique resulted in a 52% decrease in the number of *G. pallida* females [83].

Eventually, recent progresses and mechanistic insights into the molecular events between the nematodes and plants were critically reviewed. They [84,85] examined plant-PPN interactions, starting from how the nematodes perceive and find the host/non-host plant, discussing the plant’s recognition of these nematodes and the activation of its defense systems, and ending with the nematode’s response to the plant’s defenses, including failure (incompatible reaction with resistant/non-host plants) or success (compatible reaction with susceptible host plants) in overcoming the defenses [84,85]. Those authors indicated the need for more progress in biotechnology, the molecular manipulation of hosts, natural product chemistry, genome resources, and proteomics in order to translate our current knowledge into much needed ecologically benign management tactics and strategies.

## 5. Optimizing the Use of BCAs in Mechanisms Underlying Plant–PPN Interactions

BCAs may be defined in this context as organisms, e.g., microbes or bioactive compounds, that are used to control phytonematodes. Many challenges face the sound exploitation of plant-PPN interactions. Therefore, a full useful spectrum of exploiting these interactions should address the nematicidal activity of BCAs. As in the above-mentioned key disease triangle (the nematode, the host plant, and the environment), BCAs operate to the favor of the host during plant-PPN interactions. Optimizing BCAs is especially important because the performance of BCAs and/or their bioactive compounds are often criticized for being slower-acting, more inconsistent, and/or less effective than chemical nematicides [14]. Hence, each species/strain of BCA should be adapted to many biotic and abiotic factors in their surroundings to upgrade such control. On the other hand, one major technique for adding bioactive compounds could be through biofumigation, which includes the addition of mechanically chopped, mostly brassicaceous (or sometimes non-brassica) plant material to the soil for PPN control. The biofumigant impact is mainly brought about by the volatile and toxic isothiocynates (pathogen-suppressing molecules) resulting from the hydrolysis of the secondary metabolite glucosinolate found in the added plant tissues. A detailed strategy for applying brassica/non-brassica biofumigant crops, showing the mechanisms underlying nematode suppression by these plants, was recently demonstrated [2]. These tactics offer a holistic estimation of the effect of the biofumigation approach to managing PPNs in economical crops and exploring the employability of this method in integrated nematode management.

Abd-Elgawad [16] stressed that the biocontrol of PPNs using an introduced organism may not be an effective nematicide in various settings compared to that of indigenous BCA due to ecological validity comprising the edaphic and biotic factors. Primarily, the BCA introduction method, biological species/strain, time, and dose that comply with the required level of PPN control should be wisely determined to attain the best BCA(s)–nematode host matching [15,28]. Such primary tools and techniques for BCA application should best fit the properties of the targeted PPN species, habitats, and cultivated crop(s). Clearly, the degree of their fitting will be reflected in optimizing the biocontrol gains. In other words, a delicate tackling of these factors can boost the impacts of BCAs, especially alleviating or eliminating issues related to them being less effective, more inconsistent, and/or slower-acting than chemical pesticides. Therefore, using BCAs would ideally be facilitated by agricultural conservation practices to face such issues. These exercises may include adding supplementary resources, e.g., proper organic matter, enhancing the habitat quality of BCAs via certain soil amendments, and minimizing or avoiding the unfavorable impacts of incompatible pesticides. Furthermore, the bionematicidal merits should be fully exploited, e.g., they can act additively or synergistically with other agricultural practices/inputs in IPM programs [17]. Abd-Elgawad [41] reviewed promising cases where the Mi-1.2 resistance gene was reinforced against RKN infection via overexpressing certain genes of the biocontrol agent *Paecilomyces javanicus*. Likewise, such nematicidal activities with compatible chemical pesticides should operate on a wider scale [17].

Manipulating the current arsenal of BCAs is not an easy task because soil food webs, which comprise the nematodes, have a cryptic or subterranean nature. In contrast, BCAs were recently partitioned to different categories with materialized examples of the impactful usage for each category [16]. Of special interest, the single-application, co-application, sequential-application, and dual-purpose usage of these BCAs should be followed and further expanded to strengthen plant defense against pests and pathogens, especially PPN species. Although single BCA application may be impactful against certain PPN species [15], the latter three application schemes are quite useful in IPM plans. This does not negate that the application of only one species, a single BCA application, may be more impactful than co-application (e.g., Rizotec^®^ (contains only *Pochonia chlamydosporia*) vs. NemOut^®^ (comprises *Bacillus licheniformis* + *B. subtilis* + *Trichoderma longibrachiatum*)) [86]. Eventually, in order to realize such goals, it is still essential to detect more novel BCAs/their bioactive compounds and define variables that can offer insights into how soil characteristics can be adjusted to significantly elevate the biocontrol potential via conserving/favoring certain settings or BCAs [87,88,89,90,91]. For instance, soil texture and moisture [92], salinity [93], mulching [94], and pH [95] were decisive factors in modulating nematode populations directly/indirectly by impacting their hosts or natural enemies [96].

## 6. Supporting Related Techniques for PPN Control and Commercial Application

Although tremendous strides have been realized in exploiting plant–nematode interactions to upgrade safe and effective PPN control, the current challenge is to continue the pace of investigation/innovation in a period of scarce funding in terms of both governmental and private support. More extensive research should be conducted to develop and improve the processing of various nematicidal materials and techniques. In the field of biocontrol, for instance, more research is required for BCA isolation, identification, confirmation of promising nematicidal activity, mass production, formulation, packaging, transportation, shelf life, and application. Furthermore, plans should be set to cover various aspects of raising the awareness of stakeholders of the new techniques of PPN control. Such plans should comprise information days and training targeting growers and extensions to transfer technologies and methodologies. In such workshops, the principles and major components of effective IPM schemes, e.g., prophylactic production practices, crop sequences, resistant cultivars, BCA application, plant-incorporated protectants, and even genetically engineered plants should be discussed. Unfortunately, the current epidemics and wars can also be economically reflected in hindering field expansion in applying modern technologies to exploit the gains of investigating plant-PPN interactions. A suggested approach to alleviate the lack of appropriate funding is to use standardized procedures that allow future reviews to be analytical and build on them. That is because it is mostly impossible for various research groups to act together. Moreover, a main challenge to bionematicidal applications is to characterize and widely disseminate the conditions under which their use can offer cost-effective, value-added techniques to IPM. For instance, when are specific bionematicides the optimal materials in the IPM arsenal, and when can they attain safer/more impactful nematicidal activities than other tools/methods? Strategies for synergistically or additively incorporating them with chemicals or other non-chemical controls should be expanded for a wider scale. Likewise, strategies for the rational molecular control of nematodes should be materialized in wide field scales. The broad dissemination of such strategies may not only boost the effective use of the related control measurements but may also open up relevant commercial markets that currently remain underdeveloped.

In such a period of stagnant funding, two keys will lead to relevant fruitful results. These are expanded/boosted academic–industry partnerships and shifting the mindset away from the current methods that use the classical chemical nematicide model. Such partnerships and shifts are mandatory because numerous interdisciplinary challenges arise to better exploit plant-PPN interactions. Defeating such challenges requires adopting new views of interdisciplinary specialties in the related advanced branches of bioinformatics and computational and molecular biology and analyzing large-scale omics data to optimize the recent PPN control techniques. Combined operations of these disciplines may lead to a changing scope for better crop protection [97]. They can provide precise recognition of the molecular components and pathways involved in plant responses to PPN attacks/parasitism. They will certainly facilitate the use of genome editing tools in conventional plant breeding and accurately translate how gene functions are linked to phenotypic performances [98]. Admittedly, genome editing in which DNA is modified, inserted, deleted, or replaced in the PPN genome should be more cautiously and intensively used. It can insert genes to site-specific locations [98]. Ibrahim et al. [98] stated four major approaches to gene editing to enhance the worldwide breeding of cultivars/varieties resistant to RKN in a wide range of crops. The useful approaches are homologous recombination-dependent gene targeting, recombinase-mediated site-specific gene integration, nuclease-mediated site-specific genome modifications, and oligonucleotide-directed mutagenesis. Such methods are expected to participate in quick progress in harnessing the plant–nematode interaction mechanisms and improving plant resistance against PPNs. Interdisciplinary research programs can derive more sound results when the studies are ramified. For example, using BCAs as protective treatments was effective against fungal pathogens (mycologist) present in orchard soil with nematodes (nematologist) [99]. Moreover, comparing many BCA species/strains against the pest in different types of soils may require a taxonomist/molecular biologist [100]. Exploiting the outputs of these technologies on a case-by-case basis is preferable in order to thoroughly target the given variables [101]. Boosting BCA usage may also require simultaneously exploiting new application methods against both arthropod pests and PPNs, e.g., the symbiotic bacteria (species of *Photorhabdus* [102] and *Xenorhabdus* [103]) of entomopathogenic nematodes/their metabolites.

Likewise, given the substantial losses caused by PPNs, the cost of resistance durability and durability-based molecular methods are of paramount importance for plant nematology. Therefore, precise and low-cost approaches are needed to examine the evolutionary forces impacting PPN populations and the emergence of virulent nematode populations that can break plant resistance. Because PPN virulence is linked to fitness costs, susceptible plant cultivars counter-select virulent RKNs. Consequently, Nilusmas et al. [104] could distinguish optimal rotation strategies between resistant and susceptible crops to manage RKNs and enhance crop yields. Moreover, effective strategies to boost *R* gene durability under field conditions were highlighted [105]. Such applicable results should be readily available in common comparative trials and/or agricultural practices. Ultimately, the necessity of combinations of two or more of these techniques should be emphasized even more in the final evaluation.

## 7. Conclusions

In the literature, it is quite evident that PPNs are causing worldwide yield losses in many economical crops. Hence, perfections of their control methods should be further tested. Various aspects of plant–nematode interactions should be harnessed for PPN management. They should start with optimizing nematode sampling and extraction techniques to avoid misleading results and attain cost-effective and efficient IPM programs. A precise understanding of the molecular events of PPN interactions with plants will serve in developing novel approaches for PPN molecular control. The currently investigated molecular plant-PPN interactions that contribute to plant responses against nematodes such as *R* genes, RNAi, MAS, PIs, chemo-disruptive peptides, and plant-incorporated protectants are key factors to materialize this control on a wider scale for substantial increases in crop yields. Emerging approaches for PPN management and enhancing crop yields via the sequential, dual-purpose, single, and co-application of agricultural inputs, including BCAs, should be earnestly tested, especially within IPM plans. Utilizing biologicals would ideally be supported by agricultural conservation exercises to face problems occasionally related to their slow activity, inconsistency, and effectiveness against PPNs. These and other issues require adopting new views of interdisciplinary specialties in the relevant advanced disciplines to improve the current techniques for PPN control.

## Figures and Tables

**Table 1:** Molecular markers used in screening PPN resistance in some major crops *.

Crop	Nematode Species	Resistance Genes	Marker Type	References
Tomato	*Meloidogyne incognita*	*Mi 3*	RAPD and RFLP	[71]
Eggplant	*Meloidogyne javanica*	*Mi-1.2*	RT-PCR	[72]
Wheat	*Heterodera avenae*	*CreX* and *CreY*	SCAR	[73]
Pepper	*M. incognita*, *M. arenaria*, and *M. javanica*	*Me_3_* and *Me_4_*	RAPD and AFLP	[74]
Potato	*Globodera rostochinensis*	*H1*	RFLP	[75]
Soybean	*Heterodera glycines*	*Rhg1* and *Rhg4*	SNPs	[76]
Cucumber	*M. javanica*	*mj*	AFLP	[77]
Cotton	*M. incognita*	*qMi-C14*	SSR	[78]
Cotton	*Rotylenchulus reniformis*	*Ren^ari^*	SSR	[79]
Sweet potato	*Meloidogyne incognita*	*qRmi (t)*	SNPs	[70]
Peanut	*Meloidogyne arenaria*	*Rma*	CAPS, SSR, and AFLP	[80]

* Modified from [41].

## Data Availability

Not applicable.
